# Lipotoxic hepatocytes promote nonalcoholic fatty liver disease progression by delivering microRNA-9-5p and activating macrophages

**DOI:** 10.7150/ijbs.57610

**Published:** 2021-08-27

**Authors:** Hanyun Liu, Qinghui Niu, Ting Wang, Hongjing Dong, Cheng Bian

**Affiliations:** 1 Department of Infectious Diseases, The Affiliated Hospital of Qingdao University, Qingdao 266003, P.R. China; 2 Department of Liver Center, The Affiliated Hospital of Qingdao University, Qingdao 266003, P.R. China

**Keywords:** Nonalcoholic fatty liver disease, Macrophages, MicroRNA-9-5p, Extracellular vesicles, Lipotoxic hepatocytes, Transglutaminase 2

## Abstract

M1-polarized macrophages are involved in chronic inflammatory diseases, including nonalcoholic fatty liver disease (NAFLD). However, the mechanisms responsible for the activation of macrophages in NAFLD have not been fully elucidated. This study aimed at investigating the physiological mechanisms by which extracellular vesicles (EVs)-encapsulated microRNA-9-5p (miR-9-5p) derived from lipotoxic hepatocytes might activate macrophages in NALFD. After blood sample and cell collection, EVs were isolated and identified followed by co-culture with macrophages. Next, the palmitic acid-induced cell and high fat diet-induced mouse NALFD models were established to explore the *in vitro* and* in vivo* effects of EVs-loaded miR-9-5p on NAFLD as evidenced by inflammatory cell infiltration and inflammatory reactions in macrophages. Additionally, the targeting relationship between miR-9-5p and transglutaminase 2 (TGM2) was identified using dual-luciferase reporter gene assay. miR-9-5p was upregulated in the NAFLD-EVs, which promoted M1 polarization of THP-1 macrophages. Furthermore, miR-9-5p could target TGM2 to inhibit its expression. Downregulated miR-9-5p in NAFLD-EVs alleviated macrophage inflammation and M1 polarization as evidenced by reduced levels of macrophage inflammatory factors, positive rates of CD86^+^ CD11b^+^, and levels of macrophage surface markers *in vitro*. Moreover, the effect of silencing of miR-9-5p was replicated *in vivo*, supported by reductions in TG, TC, AST and ALT levels and attenuated pathological changes. Collectively, lipotoxic hepatocytes-derived EVs-loaded miR-9-5p downregulated the expression of TGM2 and facilitated M1 polarization of macrophages, thereby promoting the progression of NAFLD. This highlights a potential therapeutic target for treating NAFLD.

## Introduction

Nonalcoholic fatty liver disease (NAFLD) is characterized by the accumulation of liver fat unrelated to other causes of liver steatosis, including liver disease and excessive drinking 1. As a progressive and chronic liver disorder, the incidence of NAFLD is increasing in parallel with rising epidemic of obesity 2, and in conjunction with other associated conditions such as type 2 diabetes and advanced liver diseases including hepatic cancer 3. Some patients with NAFLD suffer from an inflammatory state called nonalcoholic steatohepatitis (NASH), which is characterized by hepatocyte damage and innate immune cell-mediated inflammation 4. M1-polarized macrophages are involved in chronic inflammatory diseases, including NAFLD 5. However, the underlying molecular mechanisms by which the factors may govern macrophage polarization and activation in NAFLD remain incompletely understood.

Extracellular vesicles (EVs) are membrane-derived vesicles, which can be secreted by different types of cells, including hepatocytes under normal and pathological conditions 6. EVs act as key regulators in intercellular communication and the development of disease via their function in transporting complex cargoes of biologically active molecules to target cells 7. Existing evidence has noted that EVs are critical regulators in the pathogenesis of NAFLD 8. Additionallty, EVs-encapsulated microRNAs (miRNAs or miRs) are implicated in the regulation of various physiological and pathophysiological processes such as NAFLD 9. miRNAs are a family of non-coding RNAs with diverse regulatory function in eukaryotic organisms; their altered expression is associated with dysregulated liver metabolism and hepatic fibrosis, injury and tumor development, rendering them therapeutic targets for the diagnosis and treatment of liver diseases, including NAFLD 10, 11. In particular, miR-9 has a confirmed role in NAFLD 12. Essandoh et al. have demonstrated that miR-9 could affect macrophage polarization to regulate the progression of inflammation-related diseases 13. In the current study, we identified a binding site of miR-9-5p in the 3'untranslated region (3'UTR) of transglutaminase 2 (TGM2) using the StarBase website (http://starbase.sysu.edu.cn/index.php). TGM2 is a multifunctional enzyme with activities in transglutaminase (TGase) crosslinking and GTP-binding-protein signaling and kinase activities that are factors in many diseases 14. It is interesting to note that TGM2 is an M2 marker that affects the extent of hepatic inflammation and liver injury in NAFLD patients 15. Although the role of miRNAs shuttled by EVs in M1 macrophage polarization has already been investigated in NAFLD, the mechanism by which lipotoxic hepatocytes-derived EVs affect M1 macrophage polarization via interplay between miR-9-5p and TGM2 is poorly understood. Hence, we performed *in vitro* and* in vivo* assays in tissue samples and experimental models, aiming to explore the critical role of the transfer of miR-9-5p *via* lipotoxic hepatocytes-derived EVs in establishing M1 macrophage polarization in NAFLD.

## Materials and Methods

### Ethics statement

The study protocol was approved by the Ethics Committee of The Affiliated Hospital of Qingdao University and was conducted strictly in accordance with the *Declaration of Helsinki.* All subjects provided written inform consent prior to enrollment. Animal experiments were approved by the Institution Animal Care and Use Committee and conducted in line with the standard of *the Guide for the Care and Use of Laboratory animals* published by US National Institutes of Health. Efforts were made to minimize the pain of the experimental animals.

### Study subjects

NAFLD tissues were collected from the 40 patients with NAFLD (31 males and 9 females) admitted in The Affiliated Hospital of Qingdao University between March 2017 and August 2018. According to the Guidelines for Diagnosis and Treatment of NAFLD (revised in 2016), the inclusion criteria were as follows: (1) clinical diagnosis: (a) no significant drinking or alcohol consumption (< 70 g/week for women, < 140 g/week for men); (b) diffuse fatty liver diagnosed by liver imaging (excluding other causes). (2) ultrasonic imaging diagnosis of liver: (a) diffusely enhanced near-field echo of the liver, with stronger echo than that of the kidney; (b) unclear structure of the intrahepatic duct; (c) progressively decreasing far-field echo of the liver area; liver exhibiting any two of the above three symptoms were judged as diffuse fatty liver. The exclusion criteria were as follows: (1) liver diseases caused by long-term drinking; (2) viral hepatitis, immune liver disease, etc.; (3) treatment with medications such as thiazides and glucocorticoids that may affect liver metabolism within a month of adminssion; (4) severe infection; (5) heart failure and arrhythmia. A total of 40 healthy individuals (29 males and 11 females) were selected as the control group. Samples of venous blood were drawn and clinical information, including age, sex, and body mass index (BMI), were recorded. Besides, levels of alanine aminotransferase (ALT), aspartate aminotransferase (AST), gammaglutamyl transpeptidase (GGT), total cholesterol (TC), triglycerides (TG), high density lipoprotein (HDL-c) and low-density lipoprotein (LDL-c) were measured in the blood samples.

### EV isolation and extraction

Samples of peripheral venous blood (10 - 20 mL) were drawn from healthy individuals and NAFLD patients after overnight fasting. The blood samples were collected into a sodium citrate tube, fully mixed for anticoagulation, and centrifuged to collect the plasma-derived EVs. Here, the samples were centrifuged at 4 ℃ and 3000 × g for 15 min to separate blood cells and platelets for isolation of the plasma, which was then centrifuged at 4 ℃ and 3000 × g for 15 min to further remove the platelets. The plasma was mixed with phosphate buffered saline (PBS) at a ratio of 1: 1, and centrifuged at 4 ℃ and 10,000 × g for 30 min to remove large vesicles and precipitates. The obtained supernatant was filtered by passage through a 0.22 μm filter and centrifuged at 100,000 × g for 70 min to separate the EVs, which were resuspended with PBS. After re-centrifugation at 4 ℃ and 100,000 × g for 70 min, the samples were resuspended using sterile PBS to obtain the EV fraction. The number and size of EVs were detected by nanoparticle tracking analysis (NTA), and the RNA and protein of the EVs were extracted.

Blood samples (1 - 2 mL) were collected from anesthetized mice by enucleation, and the following operations were performed at 4 ℃ within two hours of collection. The blood was centrifuged at 3000 × g for 15 min and the separated plasma was centrifuged at 10,000 × g for 30 min to remove large vesicles. The supernatant was filtered by passage through a 0.22 μm filter and centrifuged at 110,000 × g for 70 min, and then resuspended with PBS. After re-centrifugation at 100,000 × g for 70 min, the samples were resuspended using sterile PBS (100 μL).

### NTA

NTA was conducted using NanoSight300 (Malvern Instruments Ltd., Worcestershire, UK) according to previously described methods 16. The track of each EV in the screen was analyzed, and was automatically converted into the diameter and concentration of EVs according to the Brownian motion principle. Next, the dilution ratio was converted into the original concentration.

### Transmission electron microscopy (TEM)

The 20 μL samples of ultracentrifugated EVs was loaded on carboncoated copper grids for two min and subjected to negative staining with phosphotungstic acid solution (12501-23-4, Sigma-Aldrich Chemical Company, St Louis, MO, USA) for five min. The mesh was washed three times with PBS to remove the excess phosphotungstic acid solution and was placed on filter paper before examination using an HT7650 TEM (Hitachi, Tokyo, Japan) at 80 kV.

### Western blot analysis

Total protein was extracted and lysis buffer containing phosphatase inhibitors, protease inhibitors and phenylmethylsulfonyl fluoride (PMSF). The protein concentration was detected using a bicinchoninic acid (BCA) kit. The samples of protein (10 - 20 μg) were separated by 8 - 12% sodium dodecyl sulfate-polyacrylamide gel electrophoresis, and electrotransferred onto a polyvinylidene fluoride membrane (0.22 μm, Merck Millipore, Billerica, MA, USA). The membrane was blocked with 5% skimmed milk powder at room temperature for 1 h, and then incubated overnight at 4 °C with the following rabbit monoclonal antibodies: CD63 (1: 500, ab134045, Abcam, Cambridge, UK), tumor susceptibility gene 101 (TSG101, 1: 500, ab125011, Abcam), Alix (1 µg/mL, 1: 1000, ab76608, Abcam), anti-TGM2 antibody (1: 1000, #3557, Cell Signaling Technology, Beverly, MA, USA), glyceraldehyde-3-phosphate dehydrogenase (GAPDH, 1: 1000, #5174, CST). The membrane was then incubated with the secondary antibody peroxidase-conjugated AffiniPure Goat Anti-Rabbit Immunoglobulin G (IgG) (H+L) (#111035003, 1: 5000; Jackson ImmunoResearch, West Grove, PA, USA) for 1 h the next day. Subsequently, the membrane was added with the luminescent solution (1863097, Thermo Fisher Scientific, Waltham, MA, USA) and developed. The results were analyzed by the Image J software. The relative protein expression was expressed as the ratio of the gray value of protein to be tested to that of internal reference (GAPDH).

### Cell treatment

Human normal hepatocytes LO2 (Shanghai Cell Bank of Chinese Academy of Sciences; RRID: CVCL-6926, Shanghai, China) were cultured in Dulbecco's modified Eagle's medium (DMEM) containing 10% inactivated fetal bovine serum (FBS) (Gibco, Carlsbad, California, USA), 10 μg/mL streptomycin and 100 U/mL penicillin (Gibco), followed by incubation in an incubator (Thermo Fisher Scientific) with 5% CO_2_ at 37 ℃. When the cells had grown to confluence in the 10 cm dishes, the cells were treated with 200 and 400 μM palmitic acid (PA; P0500, Sigma-Aldrich) for 48 h to induce steatosis, whereas untreated cells served as the control.

THP-1 cells (TIB-202, ATCC, Manassas, VA, USA) were maintained in Roswell Park Memorial Institute (RPMI)-1640 medium (Gibco) supplemented with 10% inactivated FBS (Gibco), 100 U/mL penicillin, and 100 μg/mL streptomycin and incubated in an incubator (Thermo Fisher Scientific) with 5% CO_2_ at 37 ℃. Cells were passaged directly or added with an appropriate amount of fresh medium every 3 - 4 days. The resuspended cells were directly aspirated and passaged after centrifugation. THP-1 cells were differentiated to macrophages using 100 ng/mL polarized 12 myristate 13 acetate (PMA; P8139, Sigma-Aldrich) for 24 h.

### Cell lentivirus infection

According to the instructions of a lentiviral infective kit (GenePharma Co., Ltd., Suzhou, China), THP-1 cells were seeded on a 6-well plate at a density of 6.0 × 10^5^ cells/well. Next, cells were transduced with mimic-NC, miR-9-5p mimic, In-NC, and miR-9-5p inhibitor, respectively (multiplicity of infection [MOI] = 50). Cells were cultured in a 5% CO_2_ environment at 37℃ for 72 h for the subsequent experiments.

### Cell transfection

According to the instructions of Lipofectamine transfection reagent kit (Gibco), cells in the logarithmic growth phase were seeded in a 10 cm dish at a density of 5 × 10^5^ cells/well and cultured at 37 ℃ with 5% CO_2_. Upon reaching 70 - 80% cell confluence, each dish was added with 25 pmol of miR-9-5p mimic, miR-9-5p inhibitor, mimic-NC, and inhibitor-NC (GenePharma) and 10 μL transfection reagent (the final concentration of 10 pmol/mL) for transfection. Subsequently, cells were continuously cultured for 48 h for subsequent experiments.

### EV uptake assay

A PKH26 green fluorescence kit (MINI67-1KT, Sigma-Aldrich) was used to label purified EVs from human plasma or from cell supernatants. EVs were resuspended in 1 mL Diluent C solution, which was then added with 4 μL PKH67 ethanol dye solution to prepare 4 μM dye solution. The EV suspension (1 mL) was mixed with PKH26 for 5 min, and cultured with 2 mL of 1% bovine serum albumin (BSA) for 1 min to terminate staining. The labeled EVs were ultra-centrifuged at 100,000 × g for 70 min, washed using PBS, ultracentrifuged again, and resuspended in 50 μL PBS. PKH67-labeled EVs were incubated with THP-1 cells at 37 ℃ for 12 h. The cells were fixed with 4% paraformaldehyde, washed with PBS, and labeled with Phalloidin-iFluor 647 Reagent (1: 1000, ab176759, Abcam). The nuclei were stained with 4',6-diamidino-2-phenylindole (DAPI) (Sigma-Aldrich). The uptake of labeled EVs was determined using a confocal microscope (Zeiss, Heidenheim, Germany, LSM710).

### Dual-luciferase reporter gene assay

HEK293T cells (CRL1573, ATCC) were cultured in a 48-well plate for 24 h. TGM2 3'UTR wild type (wt) and mutant (mut) plasmids (Weizhen Biotechnology Co., Ltd., Shandong, China) were constructed using psicheck2 luciferase reporter plasmid (Promega, Madison, WI, USA). The luciferase reporter plasmids wt and mut were co-transfected with 50 nmol/L miR-9-5p mimic or mimic-NC mimic into HEK-293 cells. The Firefly luciferase activity and Renilla luciferase activity were measured on the microplate (Lumat LB 9508, Germany) using a dual-luciferase reporter analysis system (E1910, Promega). The ratio of Firefly and Renilla luciferase activities was regarded as the luciferase activity. The experiments were repeated three times independently.

### Flow cytometry

M1 macrophages were detected by flow cytometry. In specific, the medium was discarded, and cells were washed with PBS two times, and then detached with 0.25% trypsin. After centrifugation, the precipitate was washed with PBS two times and the cells were counted. The cell concetration was adjusted to 1 × 10^6^ cells/mL and samples then transferred into a 15 mL centrifuge tube along with 100 μL PBS containing 2% FBS. According to the kit instructions, the cells were added with specific fluorescent flow cytometry antibody APC-Cy™7 Mouse Anti-Human CD11b (560914, BD Bioscience) and PE Mouse Anti-Human CD86 (560957, BD Pharmingen) to identify M1 macrophages. The cells were incubated at 4 ℃ for 30 min in the dark, resuspended with 3 mL PBS, centrifuged, and added with 300 μL PBS buffer. In the control group, the background marker was determined by homotype monoclonal antibody. The fluorescence cells were analyzed by flow cytometry (BD FACSVerse, Becton Dickinson, Franklin Lakes, New Jersey, USA). The positive rate of surface antigen was calculated by FlowJo (LLC, Ashland, OR, USA) in units of percent.

### Isolation and quantification of RNA

The total RNA was extracted using TRIzol reagnets (15596026, Thermo Fisher Scientific). The levels of miR-9-3p, TGM2, interleukin (IL)-1β, IL-6, and tumor necrosis factor-α (TNF-α) were determined using reverse transcription quantitative polymerase chain reaction (RT-qPCR). The primer sequences are listed in Table S1. According to the kit instructions, 500 ng RNA was reversely transcribed into complementary DNA (cDNA) using the Reverse Transcription Kit (Cat#RR037A, TAKARA, Tokyo, Japan). RT-qPCR was conducted using LightCycler480 (Roche, Indianapolis, USA). β-actin was used as an internal reference for mRNA, U6 for miR-9-5p, and cel-miR-39 for plasma and plasma-EV-miR-9-5p. Fold alterations were analyzed with the 2-^∆∆Ct^ method.

### Microarray-based gene expression profiling

The expression of miR-9-5p was predicted using the RNALOCATE database (http://www.rna-society.org/rnalocate/). The target genes of miR-9-5p were predicted using the microRNA (http://www.microrna.org/), miRDIP (http://ophid.utoronto.ca/mirDIP/) and TargetScan databases (http://www.targetscan.org/). The NAFLD-related genes were obtained using the geneCards database (https://www.genecards.org/). The gene interaction network was constructed using the STRING database (https://string-db.org/). The network graph of gene interaction was constructed using cytoscape v3.7.1, and the core degree of genes in the network diagram was counted.

### Construction of NAFLD mouse models

A total of 48 specific pathogen-free (SPF) male C57BL/6J mice (aged eight weeks old; Animal Center in Shandong) were housed individually in a SPF animal laboratory at 22 - 25 ℃ and 60 - 65% humidity with a 12-hour light/dark cycle, with free access to food and water. The experiment started after one week of adaptive feeding, and the health status of mice was observed prior to the experiment. All animal were fasted for 12 h before surgery and randomized into four groups with (1) a standard diet, (2) a high-fat high-cholesterol diet, (3) a high-fat high-cholesterol diet + AAV8-NC, and (4) a high-fat high-cholesterol diet + AAV8-anti-miR-9-5p. High fat and high cholesterol diet (D12492, Shanghai QF Biosciences, Shanghai, China) was composed of 20% protein, 60% fat, and 20% carbohydrate. After 12 weeks of high-fat feeding, 100 μL AAV8-NC or AAV8-anti-miR-9-5p (10^10^ plaque forming units [PFU] /mL) were injected into the mice though tail vein, and the mice were then fed with high-fat diet for four weeks. After 16 weeks of high-fat feeding, mice were killed by anesthesia overdose and liver samples and plasma were collected.

### Measurements of biochemical markers of plasma

The blood of mice was collected by enucleation of the orbit, stood for 2 h and centrifuged at 3000 × g for 10 min to obtain the plasma. Next, the plasma-derived EVs were obtained by ultracentrifugation, and a portion of of the EVs was frozen at -80 ℃ for use in the subsequent experiments. The expression of TC (Wako, Cat#290-63701) and TG (Wako, Cat#294-65801) in mouse plasma was detected by the kits. AST and ALT activities were detected by a biochemical instrument (ADVIA 2400 Chemistry System Analyzer, Siemens, Tarrytown, NY, USA).

### Enzyme-linked immunosorbent assay (ELISA)

The expression of interleukin (IL)-1β (ELISA Kit 96T, Dakewe, Shenzhen, China), tumor necrosis factor-α (TNF-α) (ELISA Kit 96T, Dakewe) and IL-6 (ELISA Kit 96T, Dakewe) in the supernatant of THP-1 macrophages was detected. According to the kit instructions, cell supernatant (100 μL) was incubated with biotinylated antibody working solution (1: 100, 100 μL/well) for 2 h. The optical density (OD) value was measured at 450 nm, and the results were calculated by comparing with the standard and blank control.

### Histopathological examination of liver tissues

The middle lobe tissues of liver were fixed with 10% neutral paraformaldehyde, routinely dehydrated, embedded in paraffin, sectioned into 5 μm-thickness, dewaxed with xylene, and rehydrated with gradient ethanol. Three sections were randomly selected from each mouse for hematoxylin-eosin (HE) staining. The photos were taken under a light microscope to evaluate the severity of liver injury.

### Oil red O staining

The liver tissues were embedded with OCT and then cut into 5-μm-thick sections using a freezing microtome (Leica, Weztlar, Germany). The frozen sections were removed and warmed at room temperature, and immersed in distilled water to wash off the embedding agent. The sections were then rinsed with 60% isopropanol for 2 min, stained with oil red O for 2 - 5 min, and treated with 60% isopropanol under a microscope to obtain the right degree of staining. After that, the tissues were washed immediately and counterstained with hematoxylin, and red-colored lipid droplets were photographed under a light microscope (Nikon, Tokyo, Japan).

### Immunofluorescence staining

Paraffin-embedded sections were dewaxed and rehydrated to water, and subjected to antigen retrieval in ethylenediaminetetraacetic acid (EDTA) antigen repair buffer (pH = 8.0). The sections were then incubated with 5% BSA for 30 min and incubated with the primary antibodies (F4/80, 1: 400, #30325, CST, USA; CD86, 1: 200, #91882, CST) overnight at 4 °C in a wet box. The next morning, the sections were washed with PBS (pH7.4) three times (5 min/time) and then incubated with the secondary antibody (Donkey Anti-Rabbit IgG H&L (Alexa Fluor^®^647), #ab150075, Abcam) at room temperature for 50 min. After partial drying, the sections were stained with DAPI (Sigma-Aldrich) and incubated at room temperature for 10 min in the dark. After washing, the sections were added with the autofluorescence quenching agent for 5 min, washed with running water for 10 min, and dried and sealed with anti-fluorescence quenching agent. The images were observed and photographed under a fluorescence microscope.

### Statistical analysis

Data analysis was performed using the SPSS 21.0 software (IBM, Armonk, NY, USA) or Graphpad Prism8.0 (GraphPad Software, Inc., CA, USA). Measurement data are presented as mean ± standard deviation. The enumeration data were tested by Chi-squared test. Unpaired *t*-test was used for comparisons of independent samples between two groups. For multiple independent groups, one-way analysis of variance (ANOVA) with *post hoc* Tukey tests was used. Spearman's rank order correlation analysis was performed to assess the correlation between the two parameters. Values of *p* < 0.05 were considered significant for rejection of the null hypothesis.

## Results

### miR-9-5p is upregulated in the plasma-derived EVs from NAFLD patients

**Figure 1:**
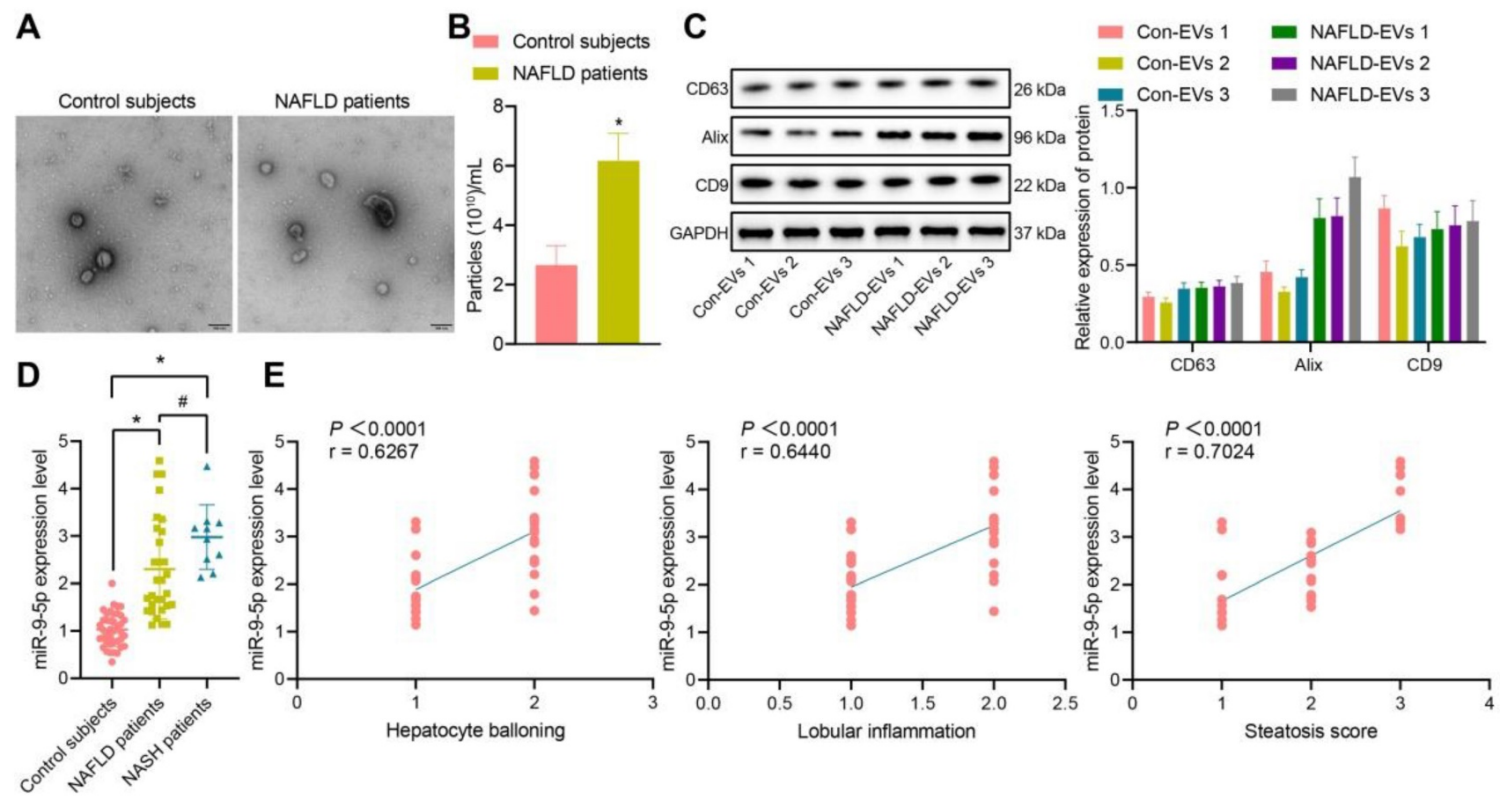
miR-9-5p expression is increased in the plasma-derived EVs from NAFLD patients. A, The morphology and diameter of EVs detected by TEM; B, The diameter and number of EVs detected by NTA. C, The expression of CD63, Alix, and CD9 in the EVs determined using Western blot analysis. D, miR-9-5p expression measured in plasma-derived EVs from NAFLD patients. E, Correlation between miR-9-5p expression and liver score of NAFLD patients. N (healthy individuals) = 24, N (NAFLD) = 11, N (NASH) = 11. * *p* < 0.05 *vs.* healthy individuals, # *p* < 0.05 *vs.* patients with NASH. Data are shown as the mean ± standard deviation of three independent experiments. Unpaired *t*-test was used for analysis differences between two groups. Data among multiple groups were compared using one-way ANOVA with Tukey *post hoc* tests used. Spearman's rank order correlation analysis was used to assess the correlation between the two parameters.

The clinical information of NAFLD patients and healthy individuals is shown in Table S2. There was no significant difference in gender and age between the healthy individuals and NAFLD patients, while BMI, AST, ALT, GGT and TG in NAFLD patients were significantly higher than those in healthy individuals. Next, addition peripheral venous blood samples were collected from 40 healthy individuals and 40 patients with NAFLD [including 10 patients with nonalcoholic steatohepatitis (NASH)] to measure the miR-9-5p level. After gradient centrifugation at low temperature, plasma-derived EVs were obtained, part of which was observed under a TEM, which showed that plasma-derived EVs were similar in structure to NAFLD-EVs (Figure 1A). The diameters of con-EVs and NAFLD-EVs were 100-200 nm, as measured by NTA (Figure 1B). Western blot analysis (Figure 1C) showed that the EVs expressed EV marker proteins. The results of RT-qPCR exhibited that the miR-9-5p level in the remaining part of plasma-derived EVs of patients with NAFLD or NASH was significantly higher than that samples from healthy individuals (Figure 1D). Based on the Spearman's rank order correlation analysis, the expression of miR-9-5p was significantly correlated with hepatocyte balloning, lobular inflammation, and Steatosis score. The expression of miR-9-5p in plasma was significantly higher in patients scored with 2-point hepatocyte ballooning and lobular inflammation than in those scored with 1-point. Meanwhile, with the increase of Steatosis score, the expression of miR-9-5p was also significantly elevated in plasma-derived EVs (Figure 1E). These results indicated that the expression of miR-9-5p in plasma-derived EVs of NAFLD patients was upregulated, and that its expression was closely related to the severity of NAFLD.

### Increased miR-9-5p level closely correlates to inflammatory cell infiltration in liver tissues of HFD-induced NAFLD mice

**Figure 2:**
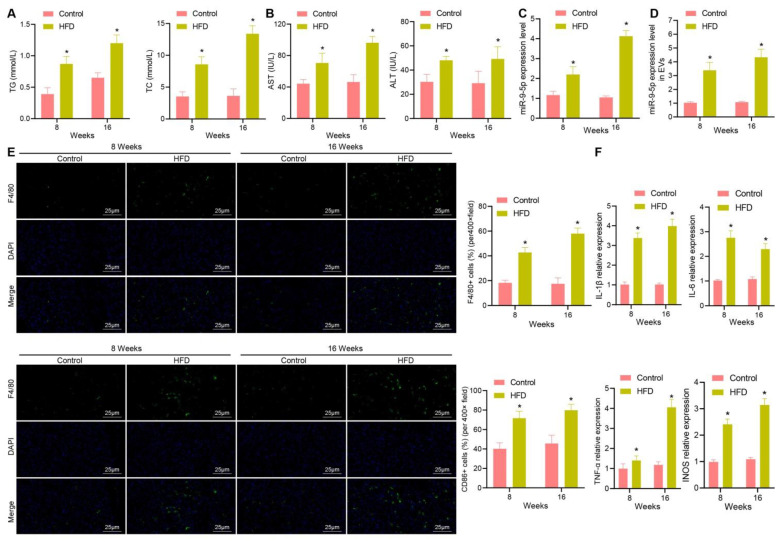
Increased miR-9-5p strongly correlates to inflammatory cell infiltration in liver tissues of HFD-fed mice. A, The expression of TG and TC in mice plasma detected by corresponding assay kits. B, The expression of AST and ALT in mouse plasma samples detected by biochemical analyzer. C, miR-9-5p level in liver tissues of HFD-fed mice measured by RT-qPCR. D, miR-9-5p level in plasma-derived EVs measured by RT-qPCR. E, The number of M1 macrophages F4/80^+^ CD86^+^ in mouse liver tissues detected by immunofluorescence staining. F, Levels of M1 macrophage surface markers in liver tissues of HFD-fed mice determined by RT-qPCR. n = 6. * *p* < 0.05 *vs.* normal mice. Data are shown as the mean ± standard deviation of three independent experiments. Unpaired *t*-test was used for analysis of differences between two groups.

In order to explore whether miR-9-5p plays an important role in the progression of NAFLD, we constructed a HFD-induced NAFLD mouse model. The expression of TG, TC, AST and ALT were increased in plasma of mice with high-fat diet for 8 and 16 weeks in varying degrees (Figure 2A, B). Next, RT-qPCR data exhibited that the expression of miR-9-5p was slightly increased in liver tissues of HFD-induced NAFLD mice at week 8 and significantly increased at week 16 (Figure 2C). Similar results were also found in plasma-derived EVs (Figure 2D). In order to explore the effect of HFD feeding on liver inflammation in mice, we undertook immunofluorescence staining, which showed that the number of F4/80^+^ CD86^+^ M1 macrophages increased significantly at week 8, and even more significantly at week 16 (Figure 2E). In accordance with the changes in the number of M1 macrophages, the expression of M1 macrophage surface markers (IL-1β, IL-6, TNF-α and inducible nitric oxide synthase [iNOS]) activity was also elevated (Figure 2F). Therefore, the obtained data suggested that the expression of miR-9-5p was increased in liver tissues of HFD-induced NAFLD mice and that this increase was strongly associated with inflammatory cell infiltration.

### PA-induced elevation of EV-miR-9-5p promotes macrophage inflammation

**Figure 3:**
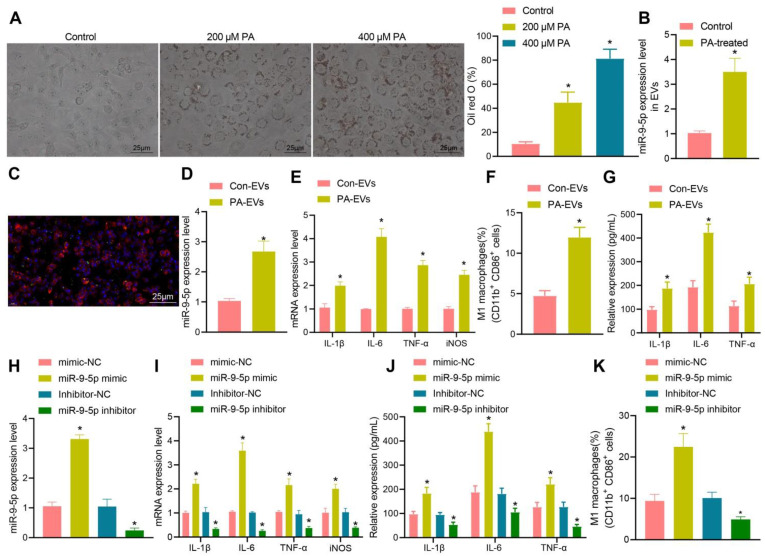
Increased miR-9-5p in hepatocyte-EVs induced by PA induces macrophage inflammation. LO2 cells were treated with different concentrations of PA (200 μM and 400 μM PA) followed by extraction of EVs. A, Lipid droplets in LO2 cells detected by Oil red O staining. B, miR-9-5p level in LO2 cells-EVs measured by RT-qPCR. C, Uptake of PKH67-labeled PA-EVs by THP-1 cells. PKH67-labeled EVs were incubated with THP-1 cells at 37 ℃ for 12 h and then observed under a fluoresence microscope. Green represents PKH67-labeled PA-EVs, blue represents DAPI-stained nuclei, and red indicates phalloidin-labeled cytoskeleton. D, miR-9-5p level in PA-EVs from hepatocytes measured by RT-qPCR. E, Levels of IL-1β, IL-6, TNF-α and iNOS in PA-EVs from hepatocytes measured by RT-qPCR. F, Positive rate of CD86^+^ CD11b^+^ elevated in PA-EVs from hepatocytes detected by flow cytometry. G, IL-1β, IL-6, TNF-α and iNOS levels in the supernatant determined with ELISA. Macrophages were treated with miR-9-5p mimic or miR-9-5p inhibitor. H, miR-9-5p level in macrophages measured by RT-qPCR. I, Levels of IL-1 β, IL-6, TNF-α and iNOS in macrophages measured by RT-qPCR. J, Expression of inflammatory factors in cell supernatant of macrophages determined by ELISA. K, Positive rate of CD86^+^ CD11b^+^ in macrophages detected by flow cytometry. N = 3. * *p* < 0.05 *vs.* LO2 cells treated with Control/mimic-NC/Inhibitor-NC. Data are shown as the mean ± standard deviation of three independent experiments. Unpaired *t*-test was used for analysis differences between two groups. Data among multiple groups were compared using one-way ANOVA with Tukey *post hoc* tests used.

To further explore the relationship between miR-9-5p and EVs in NAFLD, LO2 cells were treated with different concentrations of PA (200 and 400 μM). The results of oil red O staining (Figure 3A) showed that the number of lipid droplets was increased in LO2 cells treated with 200 μM PA, and significantly so in LO2 cells treated with 400 μM PA. Therefore, we selected the 400 μM PA treatment of LO2 cells for the subsequent experiments in whichEVs were collected from the supernatant. RT-qPCR revealed that the expression of miR-9-5p in EVs treated with 400 μM PA was significantly higher than that in untreated hepatocytes-derived EVs (Figure 3B). In order to detect the effect of EVs in PA induced hepatocytes on macrophages, we labeled EVs with PKH67 followed by co-culture of macrophages. After 12 h, the cells were stained (labeled with phalloidin) and photographed. Microscopic examination showed that a large number of EVs had entered the macrophages and distributed around the nucleus (Figure 3C). Besides, RT-qPCR determination presented that the expression of miR-9-5p was elevated in PA-induced hepatocytes-derived EVs (PA-EVs) (Figure 3D). Moreover, it was evident that PA-EVs could promote the levels of IL-1β, IL-6, TNF-α and iNOS in macrophages (Figure 3E). Flow cytometry revealed that the positive rate of CD86^+^ and CD11b^+^ was elevated in PA-EVs from the macrophages (Figure 3F). ELISA exhibited that the expression of inflammatory factors IL-1β, IL-6, TNF-α was also increased in PA-EVs (Figure 3G). Subsequently, macrophages were transduced with miR-9-5p mimic and miR-9-5p inhibitor. RT-qPCR (Figure 3H) presented that miR-9-5p level in macrophages increased significantly after infection of miR-9-5p mimic, but had decreased after miR-9-5p inhibitor treatment, validating the successful transfection efficiency. We next found that levels of IL-1β, IL-6, TNF-α in macrophages were elevated after miR-9-5p overexpression, while opposite effects were observed in response to miR-9-5p inhibitor (Figure 3I), which was also demonstrated by ELISA (Figure 3J). Flow cytometric data also showed that the positive rate of CD86^+^ and CD11b^+^ increased in macrophages transduced with miR-9-5p mimic, while the results were opposite in macrophages transduced with miR-9-5p inhibitor (Figure 3K). In conclusion, PA induction increased the expression of miR-9-5p in hepatocyte-derived EVs, which promoted macrophage inflammation.

### miR-9-5p inhibits TGM2 to induce inflammatary reaction

**Figure 4:**
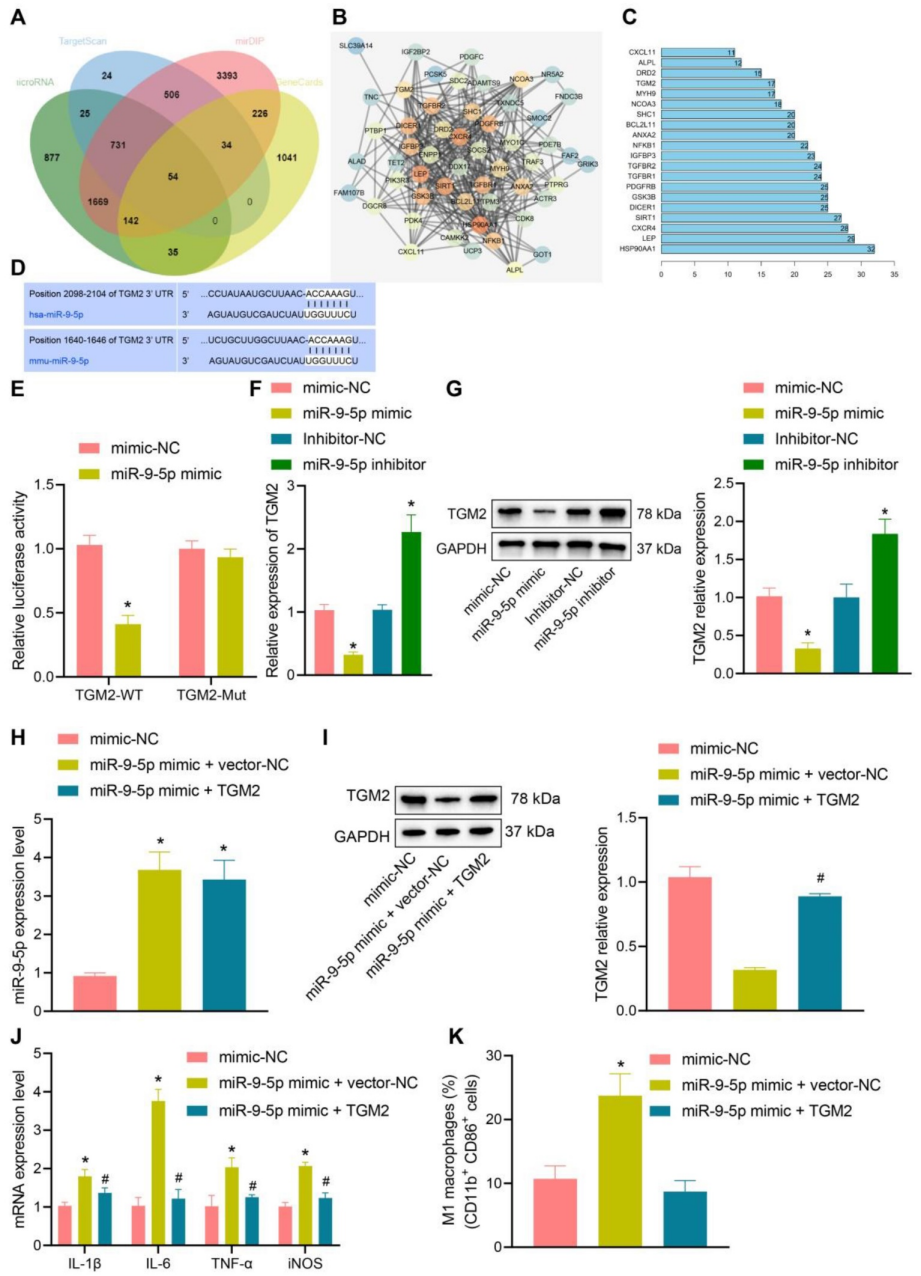
miR-9-5p induces inflammation by targeting TGM2. A, The target genes of miR-9-5p were predicted using microRNA, mirDIP, and TargetScan databases and the NAFLD-related genes were predicted using geneCards database. The middle intersection represents the intersection of four sets of data. B, The interaction network diagram of candidate target genes, each circle representes a gene, and the line between circles indicats that there was interaction between genes. More interaction gene existed in a gene indicates higher core value of the genes with core position (darker). C, The top 20 genes with the highest core value. The x-axis represents the core value, and the y-axis represents the gene name. D, The bind site of miR-9-5p at TGM2 3'UTR. E, Luciferase activity of TGM2-wt/mut detected by dual-luciferase reporter gene assay. THP-1 macrophages were transduced with miR-9-5p mimic. F, TGM2 mRNA level in THP-1 macrophages measured by RT-qPCR. G, TGM2 protein level in THP-1 macrophages measured by Western blot analysis. THP-1 macrophages transduced with miR-9-5p mimic and/or TGM2. H, miR-9-5p level in THP-1 macrophages measured by RT-qPCR. I, TGM2 protein level in THP-1 macrophages measured by Western blot analysis. J, Levels of IL-1β, IL-6, TNF-α and iNOS in THP-1 macrophages measured by RT-qPCR. K, Positive rate of CD86^+^ CD11b^+^ in THP-1 macrophages detected by flow cytometry. n = 3. * *p* < 0.05 *vs.* THP-1 macrophages transduced with mimic-NC/Inhibitor-NC, # *p* < 0.05 *vs.* THP-1 macrophages transduced with miR-9-5p mimic + vector-NC. Data are shown as the mean ± standard deviation of three independent experiments. Unpaired *t*-test was used for analysis differences between two groups. Data among multiple groups were compared using one-way ANOVA with Tukey *post hoc* tests used.

In order to further understand the effect of miR-9-5p on NAFLD, the target genes of miR-9-5p were predicted by microRNA, mirDIP, and TargetScan database. The NAFLD-related genes were searched by geneCards database, and 54 potential target genes were obtained by intersecting the retrieval results with the predicted target genes (Figure 4A). Then, the gene interaction analysis of these 54 target genes was performed, the gene interaction network diagram was constructed, and the core degree of each gene in the network diagram was counted (Figure 4B, C). In the top 20 genes with the highest core value, we identified the gene TGM2. It has been demonstrated previously that TGM2 serves as a marker of M2 macrophages, and its expression furthermore exerts effects on the progression of NAFLD and inflammatory phenotype 15, 17. Dual-luciferase reporter gene assay exhibited that luciferase activity of TGM2-wt was inhibited by miR-9-5p mimic, while no evident difference was found in TGM2-mut (Figure 4D, E), suggesting that TGM2 was a target gene of miR-9-5p. After infection of miR-9-5p into THP-1 macrophages, the expression of TGM2 was detected. The results from RT-qPCR and Western blot analysis showed that miR-9-5p overexpression could inhibit the expression of TGM2, while miR-9-5p inhibition could promote the TGM2 expression (Figure 4F, G).

To further investigate the specific protective mechanism of TGM2 in NAFLD, we overexpressed TGM2 and miR-9-5p in THP-1 macrophages. The results of RT-qPCR analysis revealed that miR-9-5p level increased in THP-1 macrophages transduced with miR-9-5p mimic and/or TGM2 (Figure 4H), and Western blot analysis presented that TGM2 expression was elevated in THP-1 macrophages transduced with miR-9-5p mimic and TGM2 (Figure 4I). Moreover, the levels of IL-1β, IL-6, and TNF-α as well as CD11b^+^ and CD86^+^ level in THP-1 macrophages transduced with both miR-9-5p mimic and TGM2 were significantly lower than corresponding levels in THP-1 macrophages transduced with miR-9-5p mimic and vector-NC (Figure 4J, K). These data suggested that miR-9-5p could inhibit the expression of TGM2 in macrophages and promote the development of inflammation. However, overexpression of TGM2 significantly reduced the proinflammatory effect of miR-9-5p on macrophages.

### Downregulated miR-9-5p in NAFLD-EVs suppresses macrophage inflammation

**Figure 5:**
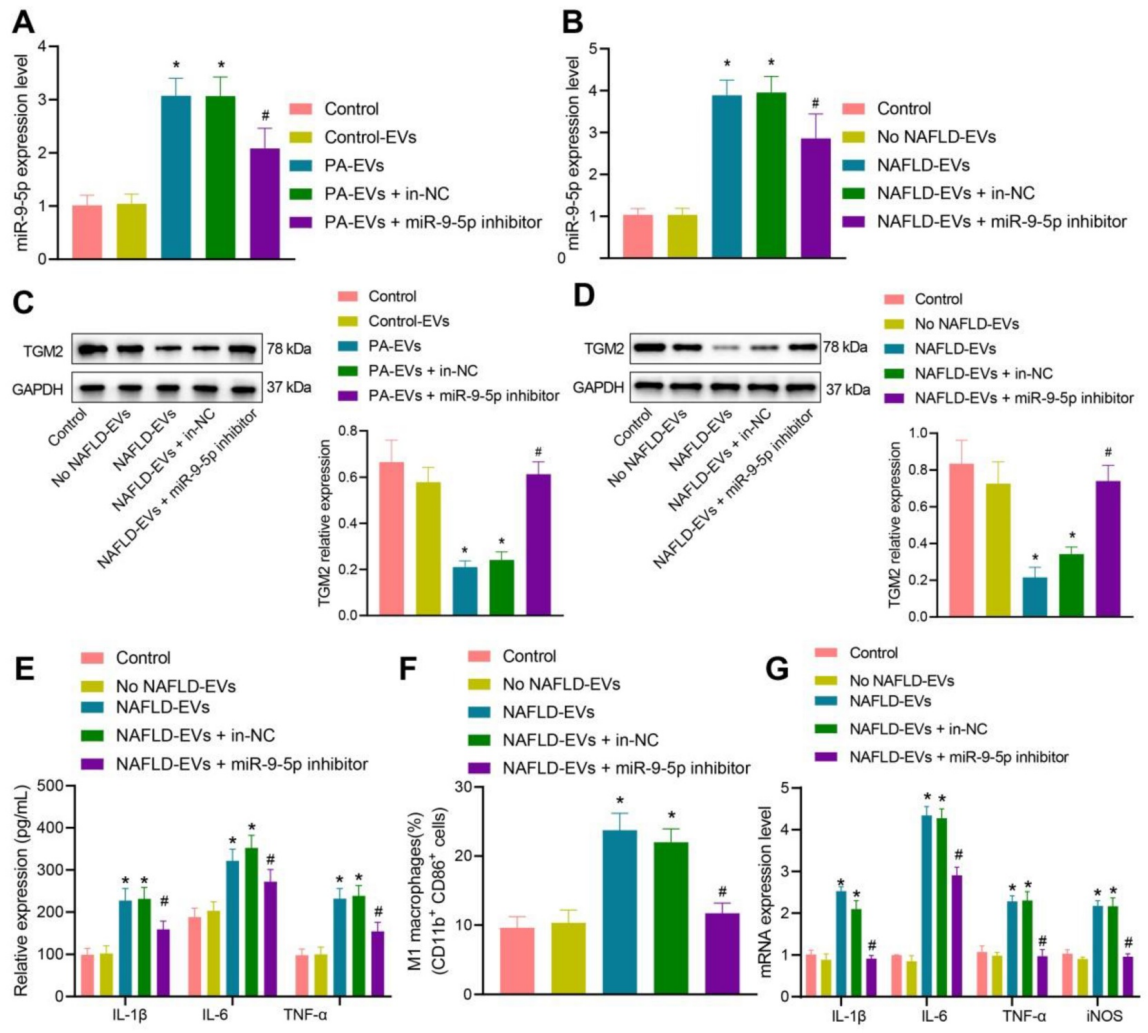
Inhibition of miR-9-5p shuttled by NAFLD-EVs relieves macrophage inflammation. Macrophages were treated with EVs from the plasma of healthy individuals or NAFLD-EVs or control-EVs and PA-EVs, and then transduced with In-NC and miR-9-5p Inhibitor. THP-1 cells were treated with 100 ng/mL PMA for 24 h to facilitate their differentiation into macrophages. PKH67-labeled EVs were incubated with THP-1 cells at 37℃ for 12 h and the following indicators were then determined: A, B, miR-9-5p level in macrophages measured by RT-qPCR. C, D, TGM2 protein level in macrophages measured by Western blot analysis. E, Levels of IL-1β, IL-6, TNF-α and iNOS in macrophages measured by RT-qPCR. F, Positive rate of CD86^+^ CD11b^+^ elevated in macrophages detected by flow cytometry. G, Expression of inflammatory factors in supernatant of macrophages determined by ELISA. N = 3. * *p* < 0.05 *vs.* macrophages transduced with Control-EVs/No NAFLD-EVs; # *p* < 0.05 *vs.* macrophages transduced with PA-EVs + in-NC/NAFLD-EVs + in-NC. Data are shown as the mean ± standard deviation of three independent experiments. Data among multiple groups were compared using one-way ANOVA with Tukey *post hoc* tests used.

To detect the inflammatory level of macrophages after inhibiting miR-9-5p expression, we conductied RT-qPCR (Figure 5A, B) and Western blot analysis (Figure 5C, D), which showed that, compared with macrophages transduced with control-EVs, miR-9-5p level increased and TGM2 protein level decreased in macrophages transduced with PA-EVs. Compared with macrophages transduced with EVs from the plasma of healthy individuals, miR-9-5p level increased and TGM2 protein level decreased after treatment of NAFLD-EVs. Compared with macrophages transduced with NAFLD-EVs + In-NC or PA-EVs + In-NC, the miR-9-5p level declined and TGM2 protein level increased in macrophages transduced with NAFLD-EVs + miR-9-5p inhibitor or PA-EVs + miR-9-5p inhibitor.

Subsequently, macrophages were treated with human plasma-derived EVs. RT-qPCR (Figure 5E), flow cytometry (Figure 5F), and ELISA (Figure 5G) presented that levels of IL-1β, IL-6, and TNF-α in the macrophages, positive rate of CD86^+^ and CD11b^+^, and levels of IL-1β, IL-6, and TNF-α in the supernatant increased in macrophages transduced with NAFLD-EVs in comparison to EVs from the plasma of healthy individuals. Compared with macrophages transduced with NAFLD-EVs + In-NC, levels of IL-1β, IL-6, and TNF-α in the macrophages, positive rate of CD86^+^ and CD11b^+^, and levels of IL-1β, IL-6, and TNF-α in the supernatant decreased in macrophages transduced with NAFLD-EVs + miR-9-5p inhibitor. The obtained data proved that plasma-derived EVs or hepatocytes-EVs containing miR-9-5p could inhibit TGM2 to induce macrophage inflammation, while inhibition of miR-9-5p suppressed the macrophage inflammation.

### Silencing of miR-9-5p ameliorates the development of NAFLD in mice

**Figure 6:**
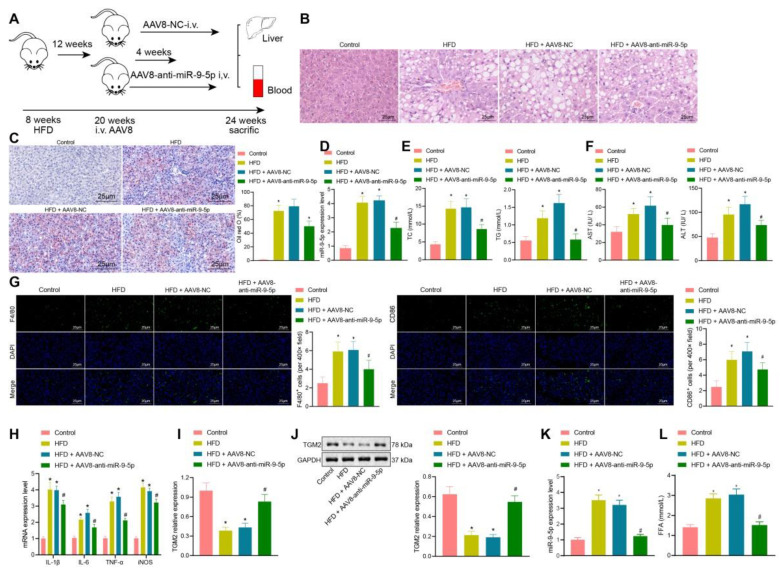
Downregulated miR-9-5p represses the occurrence and development of NAFLD *in vivo*. A, Schematic diagram of the details for HFD-induced mice injected with AAV8. B, Morphology of hepatocytes observed using HE staining. C, Lipid droplets detected by Oil red O staining. D, miR-9-5p level in liver tissues of mice determined by RT-qPCR. E, Levels of TG and TC in the plasma of mice. F, Levels of AST and ALT in the plasma of mice. G, Positive rate of F4/80 and CD86 in liver tissues of mice. H, Levels of M1 macrophage marker genes in liver tissues of mice determined by RT-qPCR. I, TGM2 mRNA level in liver tissues of mice measured by RT-qPCR. J, TGM2 protein level in liver tissues of mice measured by Western blot analysis. K, Expression of miR-9-5p in the isolated EVs from the mouse plasma detected by RT-qPCR. L, FFA determination in the mouse plasma. n = 25. * *p* < 0.05 *vs.* sham-operated mice; # *p* < 0.05 *vs.* HFD-induced mice injected with AAV8-NC. Data are shown as the mean ± standard deviation of three independent experiments. Data among multiple groups were compared using one-way ANOVA with Tukey *post hoc* tests used.

In the initial stage of the study, we had constructed a HFD NAFLD mouse model. At the 8^th^ and 16^th^ week, we found that there were different degrees of inflammatory infiltration in liver tissues, and that the number of M1 macrophages had increased, whereas the expression of miR-9-5p in liver tissues was elevated. Therefore, we further explored the effects of downregulated miR-9-5p on the degree of inflammatory infiltration in liver tissues. As shown in Figure 6A, after 12 weeks of HFD, 100 μL AAV8-NC or AAV8-anti-miR-9-5p (10^10^ PFU/mL) were injected into mice through a tail vein, and the mice were then fed with HFD for four weeks.

After 16 weeks of HFD, liver samples and plasma were collected. Observationa from HE staining (Figure 6B) showed that, compared with the normal mice (feed with a standard diet), the liver of HFD-fed mice showed obvious hepatic steatosis accompanied by abundant inflammatory cell infiltration. AAV8-anti-miR-9-5p injection significantly reduced hepatocyte edema and inflammatory cell infiltration in HFD-induced mice. Oil red O staining (Figure 6C) presented that, compared with the normal mice, there was conspicuous bright red staining and lipid droplets in liver sections from HFD-fed mice. AAV8-anti-miR-9-5p injection significantly decreased the area of Oil red O staining in HFD-fed mice. Meanshile, we found that that miR-9-5p expression and levels of TG, TC, AST, and ALT were elevated in liver tissues of HFD-fed mice in comparison to normal mice, while these effects were reversed by further injection of AAV8-anti-miR-9-5p (Figure 6D-F). Immunofluorescence staining revealed that, compared with the control fed mice, the levels of F4/80 and CD86 were increased in liver tissues of HFD-fed mice. AAV8-anti-miR-9-5p injection significantly reduced the levels of F4/80 and CD86 in liver tissues of HFD-induced mice compared with those treated by AAV8-NC, indicating that HFD suppressed the M1 polarization of macrophages (Figure 6G). RT-qPCR (Figure 6H, I) and Western blot analysis (Figure 6J) showed that the levels of IL-1β, IL-6, TNF-α, and iNOS increased and mRNA and protein levels of TGM2 decreased in liver tissues of HFD-fed mice in comparison to control mice, while opposite results were observed by the further infection of AAV8-anti-miR-9-5p. At the same time, the EVs were isolated from the plasma of mice in different groups, and the expression of miR-9-5p in the isolated EVs was detected by RT-qPCR. The results showed higher expression of miR-9-5p in the plasma-derived EVs of 16 week HFD-fed mice than in control fed mice, while opposite results were evident in liver tissues of HFD-fed mice treated with AAV8-anti-miR-9-5p in comparison to the HFD-fed mice treated with AAV8-NC (Figure 6K). Furthermore, the content of free fatty acid (FFA) was increased in varying degrees in the plasma of HFD-fed mice relative to normal mice. However, the FFA content was decreased in the plasma of HFD-fed mice treated with AAV8-anti-miR-9-5p compared with AAV8-NC-treated HFD mice (Figure 6L). Taken together, in HFD-induced NAFLD mice, increased miR-9-5p level stimulated liver macrophages to secrete inflammatory factors by reducing TGM2, resulting in M1 polarization of macrophages and promoting the occurrence and development of NAFLD.

## Discussion

NAFLD, as the leading cause of chronic liver disease, has been gradually increasing in incidence worldwide 18, 19. Activation of inflammatory M1 macrophage polarization is implicated in the development and progression of NAFLD 20. In a confirmation of our original hypothesis, we found evidence that miR-9-5p shuttled by lipotoxic hepatocytes-derived EVs could be transferred into macrophages, whereby miR-9-5p exerted inductive properties in M1 macrophage polarization to promote the progression of NAFLD through the downregulation of TGM2.

There are many miRNAs that are specifically expressed or enriched in hepatocytes, such as miR-122 and miR-194/192 10; here our initial observations revealed that miR-9-5p expression was increased in the plasma-derived EVs of NAFLD patients, and this increased expression was closely related to the individual severity of NAFLD. In line with this, miR-9 has been detected to be upregulated in L-02 cells in NAFLD 12. miR-9 inhibition induced by MCPIP1 contributes to alleviation of lipopolysaccharides (LPS)-induced liver injury in septic mice 21. Furthermore, miR-9 has a positive relationship with steatosis during NAFLD as shown by observations that overexpression of miR-9 can decrease the intracellular lipid content 12. Meanwhile, miR-9 facilitates the hepatocellular carcinoma cell proliferation and invasion *in vitro* as well as promoting tumor growth *in vivo* 22. Moreover, our present experiments demonstrated that miR-9-5p shuttled by hepatocytes-derived EVs induced inflammatory cell infiltration and inflammatory reaction, as evidenced by elevated levels of macrophage inflammatory factors, positive rates of CD86^+^ and CD11b^+^, and levels of macrophage surface markers (IL-1β, IL-6, and TNF-α), as well as levels of TG, TC, AST and ALT (*in vivo*) in the NAFLD model, thereby contributing to M1 macrophage polarization in NAFLD. It has been reported that steatotic primary hepatocytes not only release pro-inflammatory cytokines such as TNF-α, IL-6, and IL-18, but could alsoactivate hepatic expression of M1 genes such as iNOS and TNF-α in NAFLD 23. TG, TC, AST and ALT emerged as significant biomarkers for NAFLD in a large population-based study 24. Elevated miR-9-5p expression contributed to the increased levels of pro-inflammatory cytokines (IL-6 and TNF-α) 25. M1 polarization of microglia is a leading factor in the excessive secretion of pro-inflammatory factors, including TNF-α, IL-1β and IL-6, and M1 markers CD11b and CD86 26. More specifically, miR-9 is demonstrated to promote M1 polarization transcription factors, which modulates macrophage polarization in inflammation-related diseases 13. The critical role of hepatocyte-derived EVs-encapsulated miRNA in NAFLD has also been demonstrated by Liu et al., who showed that hepatocyte-derived EVs-encapsulated miR-192-5p could activate proinflammatory macrophages and promote the progression of NAFLD by regulating the Rictor/Akt/FoxO1 signaling pathway 27. The present findings supported the notion that miR-9-5p shuttled by EVs from lipotoxic hepatocytes was overexpressed and could enhance the M1 polarization of macrophages by inducing the inflammation response in NAFLD, and confirmed that silencing of miR-9-5p could alleviate inflammation and inhibit M1 polarization of macrophages, thereby suppressing the progression of NAFLD in a rodent dietary model.

NAFLD has been well established to be characterized by hepatic steatosis, which is predominantly defined as the accumulation of hepatic lipid in the liver, that is, the uptake of fatty acids and *de novo* lipogenesis surpasses the rate of fatty acid oxidation and export 28-30. Importantly, miR-9-5p has been reported to enhance lipid accumulation during preadipocyte differentiation 31. Additionally, miR-9 expression was found to be significantly increased in oleic acid-treated cells (cells were cultured with oleic acid to induce fatty changes), which also suggests that enhanced miR-9 is associated with the development of NAFLD. The aforementioned evidence provides strong evidence of an anti-steatotic effect of miR-9-5p inhibition during NAFLD.

The present study also confirmed that miR-9-5p could target TGM2 and inhibit its expression. Besides, miR-9-5p induced inflammation through decreasing the expression of TGM2 in macrophages; however, upregulation of TGM2 could reverse the promoted effects of miR-9-5p on M1 polarization of macrophages in NAFLD. TGM2 is reported to inhibit inflammatory responses in various conditions 14. A prior study has indicated that TGM2, which is an M2 marker, is downregulated in NAFLD to an extent correlating with the increased levels of the M1 markers IL-1β and IL-6, thus aggravating hepatic inflammation and liver injury in NAFLD 15. TGM2 activity is implicated in macrophage differentiation by regulating CD14 and SR-AI receptors, and depletion of TGM2 triggers a pro-inflammatory phenotype 17. Various miRNAs regulate the expression of target genes by binding to 3'UTR to degrade target mRNA or suppress translation 32. Moreover, downregulation of TG2 is reported to promote colorectal cancer cell invasion and miR-19-induced TGM2 depletion leads to induction of invasion and metastasis in colorectal cancer 33.

To sum up, HFD in mice could induce the expression of miR-9-5p and that the transfer of miR-9-5p *via* lipotoxic hepatocytes-derived EVs negatively regulated TGM2 expression to induce the M1 polarization of macrophages in cellular and rodent NAFLD models. Our findings pave the way for the development of effective therapeutic strategies for treating NAFLD by inhibiting M1 polarization of macrophages.

## Supplementary Material

Supplementary tables.
