# Supplementary material for: Lipotoxic hepatocytes promote nonalcoholic fatty liver disease progression by delivering microRNA-9-5p and activating macrophages
Source: Int J Biol Sci. 2021 Aug 27;17(14):3745–59. doi: 10.7150/ijbs.57610 (PMC8898344; doi:10.7150/ijbs.57610)
Supplement: Supplementary file 1 — Supplementary tables. [file ijbsv17p3745s1.pdf]

Table S1 Primer sequences for RT-qPCR

Target	Primer sequence
hsa/mmu-miR-9-5p RT primer	GTCGTATCCAGTGCAGGGTCCGAGGTATTCGCACTGGATACGACTCATAC
hsa/mmu-miR-9-5p	F: GCGCCGGTCTTTGGTTATCTAGCT<br>R: CAGTGCAGGGTCCGAGGTAT
hsa/mmu-U6	F: CTCGCTTCGGCAGCACA<br>R: AACGCTTCACGAATTTGCGT
U6 RT primer	AACGCTTCACGAATTTGCGT
mmu-TGM2	F: GGTAGATGAAGCCCTGTTGC<br>R: GGCCACTTCATCCTGCTCTA
hsa-TGM2	F: GGCGAACCACCTGAACAAAC<br>R: GTGTTGTTGGTGATGTGGGC
hsa-β-actin	F: CACTGTGCCCATCTACGAGG<br>R: TAATGTCACGCACGATTTCC
hsa-IL-6	F: CCTGAACCTTCCAAAGATGGC<br>R: TTCACCAGGCAAGTCTCCTCA
hsa-IL-1β	F: ATGATGGCTTATTACAGTGGCAA<br>R: GTCGGAGATTCGTAGCTGGA
hsa-TNF-α	F: CCAGGGACCTCTCTCTAATCA<br>R: TCAGCTTGAGGGTTTGCTAC
mmu-IL-β	F: TGCCACCTTTTGACAGTGATG<br>R: TTCTTGTGACCCTGAGCGAC
mmu-IL-6	F: GGGACTGATGCTGGTGACAA<br>R: ACAGGTCTGTTGGGAGTGGT
mmu-TNF-α	F: AGAAAGCATGATCCGCGAC<br>R: TTGTGAGTGTGAGGGTCTGG
mmu-iNOS	F: CGAAACGCTTCACTTCCAA<br>R: TGAGCCTATATTGCTGTGGCT

Note: RT-qPCR, reverse transcription quantitative polymerase chain reaction; miR-9-5p, microRNA-9-5p; TGM2, transglutaminase 2; IL-6, interleukin-6; IL-1β, interleukin-1β; TNF-α, tumor necrosis factor-α; iNOS, inducible nitric oxide synthase.

Table S2 The clinical information of NAFLD patients and healthy individuals

Parameters	Healthy individuals (n = 40)	NAFLD (n = 40) (Including NASH)
Age	53.80 ± 10.01	51.35 ± 8.85
Male/Female	29/11	31/9
BMI (kg/m2)*	21.96 ± 2.12	28.02 ± 2.10
AST (U/L)*	19.42 ± 2.92	34.40 ± 6.38
ALT (U/L) *	19.91 ± 2.46	46.37 ± 3.03
GGT*	19.58 ± 1.85	77.74 ± 8.93
TC (mmol/L) *	4.34 ± 0.73	5.43 ± 0.68
TG (mmol/L)*	1.23 ± 0.26	2.21 ± 0.23
HDL-C (mmol/L) *	1.29 ± 0.16	1.98 ± 0.62
LDL-C (mmol/L) *	2.53 ± 0.33	3.76 ± 0.53

Note: * $p < 0.05$ *vs.* healthy individuals. BMI, body mass index; AST, aspartate aminotransferase; ALT, alanine aminotransferase; GGT, glutamyltranspeptidase; TC, total cholesterol; TG, triglyceride; HDL-C, high density lipoprotein cholesterol; LDL-C, low density lipoprotein cholesterol.
